# Changes of equality of medical service utilization in China between 1993 and 2018: findings from six waves of nationwide household interview survey

**DOI:** 10.1186/s12939-023-01909-3

**Published:** 2023-05-23

**Authors:** Ming Wu, Ju Huang, Hongqiao Fu, Xueqin Xie, Shiyong Wu

**Affiliations:** 1 Department of Health Policy and Management, School of Public Health, Peking University, Beijing, P.R. China; 2 Institute of Medical Information, Chinese Academy of Medical Sciences and Peking Union Medical College, Beijing, P.R. China; 3 Center of Health Statistics Information, National Health Commission of the People’s Republic of China, No. 38 Beilishi Road, Beijing, 100044 P.R. China

**Keywords:** National Health Service Survey, Health care utilization, Health reform, Equity, Visiting a doctor within last two weeks, Hospital admission, Proportion of patients who should be admitted to hospital

## Abstract

**Background:**

Changes in China’s health care system in the last three decades was remarkable. The current study aims on examine the change of equality of health care utilization in mainland China based on a nationwide household interview survey.

**Methods:**

We used household interview data extracted from six waves of National Health Service Survey between 1993 and 2018. Changes of health care utilization were descripted. Equality of the utilization were examined with univariate meta-regression across urban and rural areas, socioeconomic development regions and income groups.

**Results:**

The proportion of outpatient visits within last two weeks experienced a decrease from 17.0% in 1993 to 13.0% in 2013 and bounced back to 24.0% in 2018. The age-standardized trend remained unchanged. Hospitalization in the last 12 month increased from 2.6% in 1998 to 13.8% in 2018. The perceived unmet need of hospital admission fell from 35.9% in 1998 to 21.5% in 2018. The gaps in health care utilization between urban and rural areas, across regions and by income groups have been narrowed, implying improved equality of using medical services in the last two and a half decades.

**Conclusion:**

China has experienced significant increases in health care utilization over the past 25 years. Meanwhile, the unmet needs for health care decreased remarkably and the equality of health care utilization improved significantly. These results imply significant achievements in health service accessibility in China.

## Background

Remarkable socioeconomic changes have taken place in China in the last three decades. GDP per capita has increased from US $377 in 1993 to US $12,556 in 2021. The disposable income per capita has increased from US $331 in 1993 to US $5,157 in 2021 [1]. Proportion of people aged 65 years and over has increased from 5.9% in 1993 to 14.2% in 2021 [2]. The life expectancy at birth increased from 69 in 1990 to 78 in 2020 [2].

During the same period, China’s health system has experienced significant changes as well [3–5]. Medical facilities and workforce for health have been developing rapidly. From 1993 to 2021, the number of hospitals increased from 15.4 thousands to 36.6 thousands, ward beds per 1000 population increased from 2.6 to 6.7, and the health professionals per 1000 population increased from 1.5 to 3.0 [6]. Mean while, the demand for health services is expanding and the prevalence of chronic diseases increased from 12.3% in 1993 to 34.3% in 2018 [7].

At the same time, the Chinese government started to restructure its health insurance system. Urban Employee Basic Medical Insurance (UEBMI) was established in 1998. New Rural Cooperative Medical Scheme (NRCMS) was launched in 2003 and achieved nearly universal coverage by the end of 2008. Urban Resident Basic Medical Insurance (URBMI) was established in 2007 and almost achieved universal coverage in 2010. Now China has the largest population covered by social health insurance in the world [8, 9]. The expansion of health insurance financing year by year and better financial protection played an important role in improving urban and rural residents’ affordability for health service and contributed to alleviating the problem of “seeing a doctor is expensive” [10–12].

In addition to health insurance expansion, China initiated a new round of health care system reform in March, 2009, including improving benefits of basic health insurance, promoting coverage of basic public health services, drug supply and the regulatory system reform, public hospital reform, construction of primary health care facilities, and promotion of tiered healthcare delivery system. These reform initiatives have achieved substantial progress in terms of improving access to health service, health resource allocation and the equality in health care utilization [13–15].

After decades of experiences with development of health system, crucial questions and widespread concern remain about the response to the reforms. It is critical to examine the trends in health care utilization and equality in the past 25 years that covering a series of health reforms, which could lead to a continuously comprehensive long term effect on health system. Even though most of the studies analyzed the change of health utilization in a short period (usually within five years) based on the data from healthcare provider [16–18], the data of health service demand cannot be obtained directly, and the inpatient care utilization from the provider side is usually overestimated. In addition, a few of studies analyzed the health utilization before 2013 by using the data from the National Health Service Survey (NHSS) [19, 20]. However, the literature reveals a lack of studies describing a coherent, non-fragmented analysis of China’s health care utilization and equality from 1993 to 2018 covering a series of health reforms based on the data from demanding side.

Using data from the six waves of NHSS, this study aimed to investigate changes in healthcare utilization and its equality among the Chinese population between 1993 and 2018, during which time a series of health reforms were fully initiated. A secondary aim of this study was to conduct analysis of data comparing rate of health service use and access between urban and rural population, across regions and among income subgroups. Findings from the analysis could provide important information about the long-term progress of health care system reform on improving equality between urban and rural population, across regions and among income groups at different stages of health reform. In addition, the northeast was added in the region subgroups for regional equity analysis, which provides a more refined understanding of the regional differences of healthcare utilization and a new perspective of the changes in equality.

## Methods

### Study design and data source

The dataset used in this study was household interview data that reported by residents of community dwalling from the NHSS, which was carried out by the National Health Commission (NHC) (previously named as Ministry of Health, and Health and Family Plannng Commssion) of the People’s Republic of China. It was a serial cross-sectional survey launched in 1993 with six waves of available datasets comprising the years of 1993, 1998, 2003, 2008, 2013, and 2018. It employed a multistage stratified random sampling method, involving counties, towns, and communities (villages) [19]. The NHSS is nationally representative dataset and it covered 31 provinces, autonomous regions and municipalities. In first four waves, approximately 94 counties in 31 province-level administrative units were surveryed with minor changes in sample counties/districts. Around 55 thousand households were surveyed in each wave and the sample size dropped from 214,844 individual respondents in 1993 to 177,501 in 2008. In the 2013 wave, 62 counties/districts were added to the preceding 94 sample counties and the final sample size increased to 273,688 individual respondents. The sample sites at county/district level in the 2018 NHSS remained unchanged with a final sample size of 256,304 individual respondents. Table 1 shows the sample size across six waves.

**Table 1:** Sample size in six waves of NHSS, 1993–2018

	Sample size	1993	1998	2003	2008	2013	2018
Urban	Sample counties/cities/districts	27	28	28	28	78	78
	Households	15,644	16,767	16,811	16,802	46,802	49,474
	Population	54,022	54,417	49,698	46,510	133,393	130,781
	Family size(mean)	3.4	3.2	3.0	2.8	2.9	2.6
Rural	Sample counties/cities/districts	65	67	67	66	78	78
	Households	38,766	40,177	40,212	39,654	46,811	44,602
	Population	160,822	161,089	143,991	130,991	140,295	125,523
	Family size(mean)	4.2	4.0	3.6	3.3	3.0	2.8
All	Sample counties/cities/districts	92	95	95	94	156	156
	Households	54,410	56,944	57,023	56,456	93,613	94,076
	Population	214,844	215,506	193,689	177,501	273,688	256,304
	Family size(mean)	3.9	3.8	3.4	3.1	2.9	2.7

Data source: the National Health Service Survey

### Procedures and quality control

Details of the interview procedures have been reported previously [20–22]. Briefly, well-trained household interview staff did the face-to-face interview based on a structured questionnaire. It collected extensive information about demographics, socioeconomic status, self-reported health conditions, health insurance coverage, and using medical services (both outpatient and inpatient) in given period of times. For each surveyed household, all members aged 15 years and older were interviewed, and questions about children younger than 15 years were answered by other adult family members [21]. When a respondent was absent, one of the household members within this household was asked to provide relevant information. Chinese National Bureau of Statistics provided ethics approval of the survey. All respondents knew the purpose of the survey and gave consent to be interviewed. To ensure anonymity, no names or other identifiers were used in this study.

A series of quality control measures were used in the NHSS. First, several consultation meetings for the survey design were hold and pilot surveys were conducted to improve survey design and tools. Second, all interviewers received the training to assure the consistency of survey skills. Third, the NHSS had established a standardize procedure to check the quality of data such as effectiveness, consistency, and completeness. The check results were used to guide the work of interviewers. Forth, each surveyed county had a team who was responsible for the quality of data. This team randomly selected 5% of surveyed households and reinterviewed them to check the accuracy of data collected by interviewers. If the consistency between the original survey and re-interview survey was less than 95%, all sample households in this area would be re-interviewed. Fifth, when the scheduled household was unavailable after three attempts to conduct the survey, a household in the waiting list was selected and the completion rate of the interviews should be more than 95%. Last, double data entry has been used since 2003 [20, 21].

### Statistical analysis

Three indicators were used to measure changes in healthcare utilization that self-reported by respondents of dwellings in China: outpatient care utilization rate, hospital admission rate, and the proportion of patients who had been suggested being hospitalized care and not be admitted. Specifically, in all waves of NHSS, respondents were asked how many times they visited a doctor within two weeks prior to the survey. This question was consistent across waves and were used to measure outpatient healthcare utilization from 1993 to 2018. In addition, since 1998, respondents were asked how many times have they admitted to hospital within one year prior to the survey and whether they were suggested be admitted to hospital but they were not admitted. These questions could be used to measure the self-reported hospital admission rate and the unmet inpatient care needs from 1998 to 2018 [23, 24]. While in 1993 NHSS, respondents were only asked whether they were admitted to hospital in 1992, so the frequency of hospital admissions for respondents in a year was not investigated, which is inconsistent with the other waves of NHSS. In order to comparable, the hospital admission rate and unmet hospitalization needs were calculated from 1998 to 2018. According to the age structure of 2018 National Sample Census by National Bureau of Statistics, we standardize outpatient care utilization rate and hospital admission rate.

Descriptive analysis and trend analysis were performed to examine the changes in outpatient and inpatient service utilization and residents’ unmet inpatient care needs between 1993 and 2018. The univariate Meta-regression analysis was conducted to assess the gaps between urban and rural population, across regions and by income groups and the changes in equality of health care utilization. The rural areas, northeastern regions and lowest quartile were chosen as reference groups. The heterogeneity test showed that all the indicators of each group satisfied I^2^ > 50% and *p* < 0.05, indicating a high degree of heterogeneity between groups, so the random effect model was used to perform Meta-regression.

Specifically, urban areas referred to the geographical administrative areas identified as prefecture-level cities, while rural areas referred to the geographical administrative areas identified as counties/county-level cities. We divided China into four geographic regions: eastern, central, western and northeastern, according to the classification from National Bureau of Statistics [25]. The eastern region included 10 provinces and municipalities (Beijing, Tianjin, Hebei, Jiangsu, shanghai, Zhejiang, Fujian, Guangdong, Hainan, Shandong). The central region included 6 provinces (Shanxi, Henan, Anhui, Hubei, Hunan, Jiangxi) and the western regions included 12 provinces and autonomous regions (Inner Mongolia, Guangxi, Chongqing, Sichuan, Guizhou, Yunnan, Xizang, Shaanxi, Gansu, Qinghai, Ningxia, Xinjiang). Northeastern regions included 3 provinces: Jilin, Heilongjiang, and Liaoning.

The income quartiles were constructed by household income per capita on base of respondent answer in each county. They are highest income quartile, higher income quartile, lower income quartile and lowest income quartile. In NHSS, respondents were asked whether their family listed as a local poor household or covered by subsistence allowance system, so we further divided the lowest income quartile into poverty group and non-poverty group. Poverty group included poor households and population covered by subsistence allowance system, and the rest household in the lowest income group was classified into the non-poverty group.

## Results

### Geographic and demographic characteristics of respondents

Respondents in urban areas accounted for about 25% of total respondents in the first four waves of NHSS. Sample size for urban respondent increased in the 2013 wave to reflect the population distribution in the urban and rural areas. The proportion of urban respondents outpaced its rural counterparts in the 2018 wave (Table 1). In terms of the geographic characteristics, respondents in the western region remained the largest share throughout six waves, but it presented a decline. Respondents in the eastern region had the second largest share, remaining 30% ~ 31%, while respondents in the northeastern region was the least, accounting for about 8% of total respondents. Table 2 presents geographic characteristics of respondents in six waves of NHSS. The change of urban/rural radio in respondents is typical for the total population in China that the urban population accounted for about 28% in 1993 and 62% in 2018.

**Table 2:** Geographic characteristics of survey respondents (%)

		1993	1998	2003	2008	2013	2018
Residents	Urban	25.1	25.3	25.7	26.2	48.7	51.0
	Rural	74.9	74.7	74.3	73.8	51.3	49.0
Region	East	30.7	30.5	30.8	31.3	30.1	31.3
	Central	23.7	22.3	22.3	22.5	27.8	27.0
	West	37.6	39.7	39.3	38.5	34.2	34.0
	Northeast	7.9	7.5	7.6	7.7	7.8	7.7

Data source: the National Health Service Survey

The sex ratio structure of surveyed people remained largely unchanged across six waves. The ratio of urban female was slightly higher than urban male while the condition in rural areas was opposite. As for regions, the ratio of female in eastern was slightly higher than male in five waves of NHSS (1993, 2003, 2008, 2013, 2018) while the ratio of female in other regions was slightly higher than male since 2013. Compared with the 1993 NHSS, the proportion of respondents aged below 15 years in the 2018 NHSS declined by about 10 percentage points while proportion of respondents aged above 65 years increased by 11.8 percentage points. The proportion of urban respondents aged above 65 years increased from 9.9% in 1993 to 19.3% in 2018 and its rural counterparts increased from 5.8% to 17.8% in the same period. Table 3 presents demographic characteristics of NHSS respondents.

**Table 3:** Sex and age of survey respondents (%)

		1993	1998	2003	2008	2013	2018
Urban	Sex						
	Female	51.0	50.9	51.0	51.5	51.2	51.3
	Male	49.0	49.1	49.0	48.5	48.8	48.7
	Age						
	< 5	5.4	3.6	3.4	3.5	4.8	5.7
	5 ~ 14	13.8	12.2	10.5	8.3	8.8	9.7
	15 ~ 64	70.9	71.5	71.9	71.8	70.1	65.3
	≥ 65	9.9	12.7	14.2	16.4	16.3	19.3
Rural	Sex						
	Female	49.3	48.6	49.0	49.5	49.8	50.0
	Male	50.7	51.4	51.0	50.5	50.2	50.0
	Age						
	< 5	9.1	5.8	5.3	6.1	6.3	6.2
	5 ~ 14	20.1	20.2	17.5	13.5	11.6	12.9
	15 ~ 64	65.0	66.9	69.3	70.5	68.9	63.1
	≥ 65	5.8	7.1	7.9	9.9	13.2	17.8

Data source: the National Health Service Survey

Table 4 shows the change in social health insurance coverage among surveyed people. In the NHSS, coverage rate of social health insurance increased from 20.5% in 1998 to 97.1% in 2018. Urban areas experienced an increase from 52.1% in 1998 to 96.5% in 2018, with a large rise between 2003 and 2008. Social health insurance coverage in rural areas increased from 9.9% in 1998 to 97.8% in 2018, with a large rise between 2003 and 2008. The proportion of surveyed people without health insurance coverage in 2018 was only 2.9% and 2.0% for urban and rural areas respectively.

**Table 4:** Coverage of social health insurance scheme of survey respondents (%)

	1993	1998	2003	2008	2013	2018
Urban	69.0	52.1	49.7	71.9	93.7	96.5
Rural	13.7	9.9	12.7	92.5	97.5	97.8
All	27.6	20.5	22.2	87.1	95.6	97.1

Data source: the National Health Service Survey

### Outpatient healthcare utilization

As shown in Table 5, the proportion of outpatient visits within last two weeks priot to the survey declined before 2010 and increased rapidly during the period between 2013 and 2018. It reached to 24.0% in 2018, with 25.0% in rural areas and 23.0% in urban areas. After standardization according to the age structure of 2018 National Sample Census, this rate still presented a decline between 1993 and 2013 but sharply increased between 2013 and 2018. The age-standardized rate for outpatient care utilization was still higher in rural areas than in urban areas.

**Table 5:** Proportion of outpatient visits within last two weeks, 1993–2018

	Proportion of outpatient visits within last two weeks (%)						Standardized proportion of outpatient visits within last two weeks (%)					
	1993	1998	2003	2008	2013	2018	1993	1998	2003	2008	2013	2018
Urban	19.9	16.2	11.8	12.7	13.3	23.0	24.4	18.2	11.7	11.6	11.8	19.4
Rural	16.0	16.5	13.9	15.2	12.8	25.0	19.7	19.5	15.7	15.6	11.8	21.7
East	21.4	17.6	14.9	16.9	16.5	26.1	22.5	19.1	15.3	16.3	14.3	21.9
Central	14.7	16.0	11.4	13.6	9.9	22.1	16.8	17.9	12.4	13.5	9.0	18.9
West	20.0	18.3	14.4	14.6	13.2	25.2	23.8	21.6	16.7	15.3	12.5	22.3
Northeast	14.5	11.7	7.7	7.4	10.0	16.7	16.9	13.1	8.5	7.7	9.1	14.0
Highest income	18.6	16.4	13.0	13.4	11.0	20.1	21.5	19.0	14.8	14.1	11.1	19.1
Higher income	17.1	15.5	12.5	13.3	11.9	22.2	19.3	18.0	14.1	14.0	11.4	20.1
Lower income	19.2	17.1	13.2	14.8	13.3	24.8	21.9	19.5	14.7	15.0	12.0	21.1
Lowest income	19.9	19.7	14.8	16.9	16.4	30.1	21.3	20.4	14.9	15.0	12.5	22.5
Non-poverty	-	19.2	14.6	16.9	15.7	29.0	-	19.9	14.7	15.1	11.8	21.3
Poverty	-	23.1	16.6	17.0	19.4	34.4	-	23.8	16.6	14.8	15.5	27.1
All	17.0	16.4	13.4	14.5	13.0	24.0	21.1	19.3	14.7	14.6	11.8	20.5

Data source: the National Health Service Survey

Poverty group included poor households and population covered by subsistence allowance system, the rest household in the lowest income group was classified into the non-poverty group

The changes in outpatient utilization for urban and rural areas were similar with the overall trend. The outpatient utilization for rural areas exceeded its urban counterparts after 1998 and their gap had been reduced by 2018. As for regions, the proportion of outpatient visits within last two weeks in eastern and western provinces was relatively higher than central and northeastern provinces. The gap between the eastern and the northeastern region in 2018 was as large as 8 percentage points after taking age structure into consideration. As for trends, all regions experienced a decline in the proportion of outpatient visits within last two weeks between 1993 and 2003, with the largest drop of 8 percentage points in the northeastern region after age structure being considered. However, the proportion of outpatient visits within last two weeks experienced a rise in all regions between 2008 and 2018. In spite that the rise of northeastern regions in this period was the largest, the proportion of outpatient visits within last two weeks in northeastern regions was only 16.7% in 2018 and remained the lowest. The highest counterpart was the eastern region, reaching 26.1% in 2018.

The age-standardized proportion of outpatient visits within last two weeks for all income groups declined between 1993 and 2003. This rate then climbed up since 2008 for all income groups. The largest rise occurred in the poverty group, increased 12 percentage points. The smallest rise occurred in the highest income group, increased 6 percentage points. After age structure being considered, the proportion of outpatient visits within last two weeks for lowest income group was 3 percentage points higher than highest income group, while the poverty group was 8 percentage points higher than highest income group.

### Inpatient care utilization

As shown in Table 6, the hospital admission rate displayed a gradual rise between 1998 and 2018, increased from 2.6% to 13.8%. The age-standardized hospital admission rate experienced a 3-times increase to 12.1% in 2018 compared with 1998. In this period, the most rapid growth of hospital admission rate occurred between 2013 and 2018, with an increase of about 4 percentage points.

**Table 6:** Hospital admission rate, 1998–2018

	Hospital admission rate (%)					Standardized hospital admission rate (%)				
	1998	2003	2008	2013	2018	1998	2003	2008	2013	2018
Urban	3.6	4.2	7.1	9.1	12.9	3.7	4.1	6.2	8.0	11.0
Rural	2.3	3.4	6.8	9.0	14.7	2.7	3.8	7.2	8.7	13.3
East	2.2	3.5	6.2	7.8	11.2	2.4	3.6	6.0	6.9	9.7
Central	2.9	3.3	7.3	9.4	15.1	3.4	3.7	7.6	8.7	13.0
West	2.8	3.9	7.2	10.0	15.3	3.4	4.4	7.6	9.7	14.1
Northeast	2.7	3.4	6.3	8.5	12.4	3.2	3.5	6.5	7.4	10.1
Highest income	2.9	4.2	7.3	8.3	12.6	3.6	4.8	7.9	8.5	12.3
Higher income	2.4	3.5	6.7	8.7	13.1	2.9	4.0	7.2	8.6	12.2
Lower income	2.5	3.2	6.4	8.9	13.4	3.0	3.6	6.6	8.3	11.8
Lowest income	2.7	3.4	7.0	10.4	16.5	2.8	3.5	6.4	8.2	12.4
Non-poverty	2.6	3.4	6.9	9.9	15.0	2.7	3.4	6.3	7.8	11.2
Poverty	4.1	4.1	7.5	12.4	22.3	4.5	4.4	6.5	9.9	16.9
All	2.6	3.6	6.8	9.0	13.8	3.1	4.0	7.1	8.4	12.1

The frequency of hospital admissions in a year was not investigated in 1993 NHSS, which is inconsistent with the other waves of NHSS, so the hospital admission rate was calculated from 1998 to 2018

Data source: the National Health Service Survey

Poverty group included poor households and population covered by subsistence allowance system, the rest household in the lowest income group was classified into the non-poverty group

The hospital admission rate in urban and rural areas also displayed a gradual rise between 1998 and 2018 and the gap between them had narrowed down. The age-standardized hospital admission rate in urban areas was slightly higher than rural areas between 1998 and 2003, but was outpaced by rural areas since 2008. Their gap gradually expanded since then. The hospital admission rate in rural areas was 14.7% in 2018, exceeding urban areas in the same year.

The hospital admission rate across regions displayed a gradual rise between 1998 and 2018. The hospital admission rate in the eastern region remained the lowest (2.2% ~ 11.2%) while that in the western region remained relatively high (2.8% ~ 15.3%). The gap of age-standardized hospital admission rate between eastern and western regions extended from 1 percentage point in 1998 to 4 percentage points in 2018. The growth rate of western regions exceeded eastern regions in all periods.

The hospital admission rate across income groups displayed a gradual rise between 1998 and 2018. The age-standardized hospital admission rate in highest income group was higher than other groups (except 2018). The age-standardized hospital admission rate in the poverty group reached its peak to 16.9%, 4 percentage points higher than highest income group.

### Unmet need of hospitalization

As shown in Table 7, the unmet need of hospital admission displayed a decline between 1998 and 2018, dropping from 35.9% in 1998 to 21.5% in 2018. The extent of decrease between 1998 and 2008 was larger than that between 2008 and 2018.

**Table 7:** Unmet need of hospitalization 1998–2018

	Proportion of unmet need of inpatient medical care (%)				
	1998	2003	2008	2013	2018
Urban	30.4	26.1	25.0	17.7	21.0
Rural	38.5	28.1	22.6	16.8	21.9
East	32.7	23.2	19.9	13.5	16.2
Central	36.2	29.1	23.5	17.9	21.0
West	36.8	28.8	23.7	17.9	23.3
Northeast	39.7	32.1	31.0	23.5	30.4
Highest income	27.6	18.7	15.6	13.2	15.6
Higher income	32.2	22.6	19.4	14.5	19.2
Lower income	36.9	28.2	24.2	17.5	23.4
Lowest income	46.4	39.7	34.5	22.9	26.7
Non-poverty	44.1	37.9	31.1	20.6	25.5
Poverty	55.4	51.1	45.6	29.7	30.0
All	35.9	27.5	23.2	17.2	21.5

Indicators above were calculated by the number of people

Poverty group included poor households and population covered by subsistence allowance system, the rest household in the lowest income group was classified into the non-poverty group

In most years except 2008, the unmet need of hospital admission in urban areas was lower than rural areas. As for regions, the unmet need of hospital admission was lower in eastern regions but was higher in northeastern regions, with a gap of 14 percentage points. All regions experienced a sharp decline in the unmet need of hospital admission, with a larger fall in central and eastern regions but a smaller fall in the northeastern region.

The unmet need of hospital admission varied across income groups. The rate in the wealthiest quartile was the lowest and the rate in the poorest quartile was the highest. However, unmet need of hospital admission displayed a decline in all income quartiles between 1998 and 2018, and the gap between the wealthiest quartile and the poorest quartile shrank year by year, with a decrease from 19 percentage points in 1998 to 14 percentage points in 2018. The rates for the wealthiest quartile and the poorest quartile were 15.6% and 30.0% in 2018, respectively.

### Equality of health care utilization

Table 8 reports the results of univariate Meta-regression analysis on outpatient visits and hospital admission. The odd ratio (OR) for age-standardized proportion of outpatient visits within last two weeks between urban and rural areas declined from 1.32 (95%CI: 1.29–1.35) in 1993 to 0.92 (95%CI: 0.9–0.94) in 1998, implying that the outpatient utilization in urban areas was 1.32 times of that in rural areas in 1993 and decreased to 0.92 times in 1998. In spite of some fluctuations, OR had been less than 1 since 2003, implying that the age-standardized proportion of outpatient visits within last two weeks in rural areas was higher than urban areas. It mirrored the improvement in equality of outpatient service utilization in urban and rural areas.

**Table 8:** Univariate Meta-regression analysis on age-standardized rates of outpatient and inpatient care utilization, 1993–2018

	OR(95%CI)					
	1993	1998	2003	2008	2013	2018
**Urban and rural (comparator: rural)**						
Outpatient visit rate	1.32(1.29–1.35)	0.92(0.9–0.94)	0.71(0.69–0.73)	0.71(0.69–0.73)	1.00(0.98–1.02)	0.87(0.85–0.89)
Hospital admission rate		1.28(1.13–1.45)	0.8(0.69–0.93)	0.64(0.57–0.72)	0.92(0.85–0.99)	0.73(0.7–0.77)
**Regions (comparator: northeast)**						
Outpatient visit rate						
East	1.43(1.37–1.49)	1.57(1.49–1.65)	1.95(1.83–2.07)	2.33(2.18–2.49)	1.67(1.58–1.75)	1.72(1.65–1.80)
Central	0.99(0.95–1.04)	1.45(1.37–1.52)	1.52(1.43–1.50)	1.87(1.75–2.00)	0.99(0.94–1.04)	1.43(1.37–1.50)
West	1.54(1.47–1.60)	1.83(1.74–1.92)	2.16(2.03–2.29)	2.16(2.03–2.31)	1.43(1.36–1.50)	1.76(1.69–1.84)
Hospital admission rate						
East		1.10(0.84–1.43)	1.85(1.35–2.54)	1.95(1.52–2.51)	1.48(1.23–1.76)	1.51(1.33–1.72)
Central		1.46(1.12–1.91)	1.54(1.11–2.14)	2.05(1.59–2.64)	1.17(0.97–1.40)	1.75(1.54–1.99)
West		1.77(1.37–2.27)	2.48(1.82–3.37)	2.32(1.82–3.37)	1.82(1.53–2.16)	2.25(1.99–2.54)
**Income(comparator: lowest income)**						
Outpatient visit rate						
Highest income	1.10(1.06–1.13)	0.99(0.96–1.02)	1.00(0.97–1.04)	0.97(0.94–1.01)	0.87(0.85–0.90)	0.80(0.78–0.83)
Higher income	0.88(0.86–0.91)	0.86(0.83–0.88)	0.90(0.87–0.93)	0.92(0.89–0.96)	0.90(0.87–0.93)	0.87(0.84–0.89)
Lower income	0.96(0.93–0.99)	0.87(0.85–0.90)	0.97(0.94–1.01)	0.96(0.92–0.99)	0.95(0.92–0.99)	0.93(0.91–0.96)
Hospital admission rate						
Highest income		1.28(1.09–1.49)	1.38(1.17–1.63)	1.21(1.06–1.38)	0.92(0.82–1.03)	0.83(0.77–0.89)
Higher income		0.91(0.91–1.29)	1.09(0.91–1.29)	1.05(0.91–1.20)	0.96(0.86–1.07)	0.88(0.82–0.94)
Lower income		0.96(0.82–1.13)	1.01(0.84–1.20)	0.99(0.86–1.14)	0.97(0.87–1.08)	0.90(0.84–0.97)

The frequency of hospital admissions in a year was not investigated in 1993 NHSS, which is inconsistent with the other waves of NHSS, so the hospital admission rate was calculated from 1998 to 2018

The OR for age-standardized hospital admission rate between urban and rural areas kept declining from 1.28 (95%CI: 1.13–1.45) in 1998 to 0.73 (95%CI: 0.7–0.77) in 2018. In spite of some fluctuation, OR had been less than 1 since 2003, implying that the age-standardized hospital admission rate in rural areas was higher than urban areas. This may be largely attributed to the coverage expansion of NRCMS from 2003 to 2008 and URBMI from 2007 to 2010, as well as the gap of reimbursement rate between urban and rural areas.

Compared with northeastern regions, the OR of age-standardized proportion of outpatient visits within last two weeks experienced a rise at early stage and then decline afterwards. It implied the gap in outpatient service utilization between northeastern regions and other regions enlarged. However, as suggested by the similar ORs in Table 8, the gap across eastern, central and western regions had been narrowed gradually, especially for the gap between eastern and western regions.

The age-standardized hospital admission rate in northeastern regions was significantly lower than other regions. OR of hospital admission rate also experienced a rise with increases at early stage and then decline afterwards. Although the inpatient reimbursement rate in northeastern regions has increased much since 2008 and its gap in inpatient reimbursement rate with other regions kept reducing, the gap in inpatient service utilization between northeastern regions and other regions was extending. Gap between eastern regions and central regions was shrinking while gap between eastern regions and western regions remained roughly unchanged.

OR of age-standardized outpatient healthcare utilization by income groups for different years was roughly unchanged. The gap in this indicator between best-off quartile and poorest quartile was continuously shrinking, with OR falling from 1.10 (95%CI: 1.06–1.13) in 1998 to 0.80 (95%CI: 0.78–0.83) in 2018. OR of different income quartiles was approaching to each other, suggesting the gap in outpatient service utilization across income quartiles was narrowing down.

OR of age-standardized hospital admission rate by income groups in different years displayed a decline. Except wealthiest quartile, OR for other income quartiles remained steady with time. The gap in age-standardized hospital admission rate between best-off and the poorest quartiles was shrinking year by year, with OR falling from 1.28 (95%CI: 1.09–1.49) in 1998 to 0.83 (95%CI: 0.77–0.89) in 2018. OR of different income quartiles was approaching to each other, suggesting that the gap in inpatient service utilization across income quartiles was narrowing down. It may owe to the shrinking gap in inpatient reimbursement rate across different income quartiles. The gap between best-off quartile and poorest quartile fell from 40.5 percentage points in 1998 to 5.3 percentage points in 2018.

## Discussion

From 1993 to 2008, the probability of feeling sick within two weeks increased from 13.7% to 17.6% [7], while the outpatient healthcare utilization (the proportion of outpatient visits within last two weeks) showed a trend of slightly fluctuating decline, and the trends for urban and rural areas were similar with the overall trend. The NHSS data showed that from 1993 to 2008, the proportion of self-treatment increased from 20.1% to 35.7%, and the proportion of unmet treatment needs for the disease due to financial burden also increased from 33.5% to 38.2%, and declined to 27.1% and 29.2% in 2008 respectively. However, the outpatient healthcare utilization rate for the population with and without social health insurance also were similar with the overall trend, which dropped from 20.2% and 18.1% to 15.1% and 10.7%, respectively. It suggested that the social health insurance didn’t influence the outpatient healthcare utilization in this period. During this period, the health reforms can be divided into period of introducing market forces in health sector in 1990s with UEBMI established in 1998 and the period of starting restructure the medical insurance system from 2003 to 2008 with a pilot of NRCMS in 2003, and a pilot of URBMI in 2007. Actually, with economic development, allocation of market adjusting resources solved the problems of difficult access to doctors due to the shortage of medical resources. However, as a result, the lack of effective constraints on the rapidly growing supply of medical services, and in the initial stage of establishing a universal medical insurance system, with a not high level of financing and financial protect, NRCMS and URBMI mainly focusing on reimbursement for inpatient, finally increased barriers to outpatient healthcare utilization.

Our results showed that the outpatient healthcare utilization (the proportion of outpatient visits within two weeks) went up after 2008. The trend was consistent with the considerable rise of the probability of feeling sick within two weeks since 2008, which increased from 17.6% in 2008 to 33.4% in 2018. Major causes for visiting a doctor within two weeks were having cold (including acute nasopharyngitis, acute upper respiratory infection and influenza) and gastroenteritis in 1993, accounting for 48% of all visits, while hypertension and diabetes only accounted for 2.6% in the same period. The proportion of visiting a doctor within two weeks due to hypertension and diabetes increased year by year after 2003 and raised up significantly after 2008, increasing from 10.5% in 2008 to 25% in 2018. Hypertension and diabetes have become the second and third major disease for visiting a doctor following cold [13]. Trends in these increases between urban and rural areas were fairly similar, and the growth of the probability of feeling sick within two weeks in rural areas (with 16 percentage points growth) is higher than that in urban area (with 14 percentage points growth) from 2008 to 2018 [7]. In 2009, the National Basic Public Health Services Programme was implemented and hypertension, diabetes screening and management could be provided to all residents free of charge. As can be seen, the increases of outpatient healthcare utilization may largely benefit from the expansion of basic public health service and improved health management on chronic diseases since 2009 [26]. The basic public health service programme promotes the prevention and management of diseases, screens out more patients with chronic disease, which increase the demand for health services and expand the outpatient healthcare utilization. More importantly, it promotes the improvement of the accessibility of health services, making positive changes to healthcare utilization which is changed from ignorance of health condition and disease prevention, failure to diagnose and treatment, to concern about health.

Hospital admission rate increased from 2.6% in 1998 to 13.8% in 2018. The growth of age-standardized result reached to 9 percentage points, with 11 percentage points in rural area and 7 percentage points in urban area. This is a significant increase of respondent self-reported hospitalization. According to the data of the National Health Interview Survey in United States, the hospital stay in the past year decreased from 7.6% in 1998 to 7.3% in 2018 [27]. And the growth of hospital admission rate of hospital statistics data from 1998 to 2018 in Australia, Germany, Japan, the South Korea is about 2, 6, 3, 9 percentage points [28]. Compared with 2003, hospital admission rate in rural areas has a significant increase in 2008, with age-standardized annual growth rate of 13.6%. With the increase of income and availability increase of hospitalized service, it should be noted that the increase in hospital admission rate in rural areas is equivalent to the achievement of universal coverage of NRCMS in the same year and the rise of its financing level year by year [29]. The unmet need of hospital admission in urban and rural areas decreased significantly and the unmet healthcare needs due to financial burden exhibited a decline as well [13], falling from 18.3% (urban) and 24.5% (rural) in 1998 to 9.0% (urban) and 10.2% (rural) in 2018, respectively. The results showed a great consistency with the timeline of the establishment of NRCMS and URBMI, reflecting the role of social health insurance in increasing healthcare access in China [15, 30].

Why the annual hospitalization rate became higher for rural population than for urban population? In 1998, the inpatient reimbursement rate for urban and rural population was 55.9% and 9.6%, and cost for US $487.6 and $185.0 per admission, respectively. Since 2003, the NRCMS and URBMI for rural and urban areas has been successively established and the level of financing has increased year by year, then the reimbursement ratio for hospitalization expenses has also been continuously increased. In 2018, the inpatient reimbursement rate for urban and rural population reached about 57% and 53% [7], respectively. In particular, the inpatient reimbursement rate of URBMI and NRCMS increased from 35 and 26% in 2008 to 52% and 50% in 2018, significantly shrinking of differences with reimbursement rate of UEBMI. Moreover, NRCMS focuses on reimbursement for inpatient and catastrophic outpatient expenses, which to a certain extent encourages rural residents to have more inpatient services than outpatient services, and promote the large growth of inpatient care utilization for rual polulation. In addition, under the condition that the outpatient healthcare utilization for urban and rural residents in 2018 tended to be the same, the hospital admission rate of cold for rural residents in 2018 reached 11.6‰, which was much higher than that of urban residents (6.1‰), indicating that unnecessary hospitalization in rural areas may be over the city.

Aside from the northeast region, the gap of health care utilization between urban and rural areas and across regions and income groups has narrowed down and equality has been significantly improved. The utilization of both outpatient and inpatient care for rural residents and low income group experienced a considerable rise and even outpaced its counterparts in urban areas and high income groups. On the one hand, these results were related to the social-economic development and the increase of residents’ income level. On the other hand, the significant increase of health care utilization after 2003 was largely attributed to the establishment of urban and rural health insurance schemes, the shrinking of differences in inpatient reimbursement rate, the increasing demand for health services, implying the achievement of China’s basic health insurance scheme. Lagged economic development in northeast regions impacted its increasing pace of health care utilization.

In summary, the 1993–2013 and 2013–2018 results show differential changes in outpatient care utilization. The inpatient care utilization has been rising for about three decades and the gaps in health care utilization between urban and rural areas, across regions and by income groups have been narrowed. It is important to note that although the accessibility of health services has been greatly improved in recent ten years, China’s health care system still faced great challenges by the increased trend in healthcare utilization mainly caused by increasing prevalence of chronic diseases [31–33] and the prevention and management of diseases screening out more patients with chronic diseases and has large rooms to improve the accessibility and equity of health services.

### Limitations

First, as with all self-reported data from NHSS, recall bias cannot be excluded. However, the effect could be less important when comparing relative changes across waves and assuming the degree of the bias remained similar across waves. Second, the NHSS data collected every five years, which could be consistency across waves for the measurement bias. Finally, this study mainly focused on the trends in healthcare utilization, and the interpretation of changes is mainly based on the evidence of correlation analysis rather than causal association. The interpretation and attribution over the changes need to be further investigated, as nature of the current study is descriptive. However, the main utilization indicators of the six waves of NHSS have maintained a good consistency, and the current setting of our study is able to observe the changes in China’s health care utilization and equality in a long period from the perspective of demanding side, which could provide referencable evidence for informing health care planning in the future. Data collected through household interview survey is one of lens of observing equality. It could also be triangulated with other sources of data (such as clinical administrative data, or health statistic reporting data) for comprehensive understanding of equality of using medical service.

## Conclusion

China has experienced significant increases in health care utilization over the last 25 years. Annual Hospital admission rate increased year by year and the share of population with the unmet needs for health care decreased remarkably. Meanwhile, the gaps in health care utilization between urban and rural areas, across regions and by income groups have been narrowed, implying that equality has been improved. These results imply great achievements in better healthcare access in China. These achievements are closely related to the expansion of social health insurance schemes, better financing protection, the shrinking of the gap in inpatient reimbursement rate across regions and income groups, as well as improved chronic disease management due to China’s health care reform.

## Data Availability

Please contact the authors for data request.

## Abbreviations

UEBMI: Urban Employee Basic Medical Insurance
NRCMS: New Rural Cooperative Medical Scheme
URBMI: Urban Resident Basic Medical Insurance
NHSS: National Health Service Survey
NHC: National Health Commission
CI: Confidence interval
OR: Odds ratio

## References

[1] World Bank. China Data. World Bank; 2023. https://data.worldbank.org/country/china?view=chart. Accessed 11 Feb 2023.

[2] National Bureau of Statistics. China statistical yearbook 2022. National Bureau of Statistics; 2022. www.stats.gov.cn/tjsj/ndsj/2022/indexeh.htm. Accessed 11 Feb 2023.

[3] Yip WC-M, Hsiao WC, Chen W (2012). Early Appraisal of China’s Huge and Complex Health Care Reforms. Lancet..

[4] Wagstaff A, Yip W, Lindelow M (2009). China’s Health System and Its Reform: A Review of Recent Studies. Health Econ.

[5] Yip W, Hsiao WC (2008). The Chinese Health System at a Crossroads. Health Aff (Millwood).

[6] National Health Commission of the PRC. 2009 China Health Statistics Yearbook. National Health Commission of the PRC; 2009. www.nhc.gov.cn/mohwsbwstjxxzx/s7967/201307/021ac6bb4dc9484b8f8d7af130c22c28.shtml. Accessed 11 Feb 2023.

[7] Centerfor Health Statistics and Information, National Health Commission of the PRC (2018). An Anlysis Report of National Health Services Survey in China.

[8] Dong W, Zwi AB, Bai R, Shen C, Gao J (2021). Benefit of China's Social Health Insurance Schemes: Trend Analysis and Associated Factors Since Health Reform. Int J Environ Res Public Health.

[9] Jamal MH, Abdul Aziz AF, Aizuddin AN, Aljunid SM (2022). Successes and obstacles in implementing social health insurance in developing and middle-income countries: A scoping review of 5-year recent literatures. Front Public Health..

[10] Meng Q, Fang H, Liu X, Yuan B, Xu J (2015). Consolidating the social health insurance schemes in China: towards an equitable and efficient health system. Lancet.

[11] Hong L, Zhong Z (2014). Does health insurance matter? Evidence from China’s urban resident basic medical insurance. J Comp Econ.

[12] Cheng L, Liu H, Zhang Y (2015). The impact of health insurance on health outcomes and spending of the elderly: evidence from China's New Cooperative Medical Scheme. Health Econ.

[13] Ta Y, Zhu Y, Fu H (2020). Trends in access to health services, financial protection and satisfaction between 2010 and 2016: Has China achieved the goals of its health system reform?. Soc Sci Med..

[14] Tao W, Zeng Z, Dang H (2020). Towards universal health coverage: lessons from 10 years of healthcare reform in China. BMJ Glob Health..

[15] Yip W, Fu H, Chen AT (2019). 10 years of health-care reform in China: progress and gaps in Universal Health Coverage. Lancet.

[16] Dai G, Li R, Ma S (2022). Research on the equity of health resource allocation in TCM hospitals in China based on the Gini coefficient and agglomeration degree: 2009–2018. Int J Equity Health.

[17] Li Q, Wei J, Jiang F (2020). Equity and efficiency of health care resource allocation in Jiangsu Province, China. Int J Equity Health.

[18] Zhang Y, Wang Q, Jiang T (2018). Equity and efficiency of primary health care resource allocation in mainland China. Int J Equity Health.

[19] Wang S, Xie X, Zhang Y (2021). Review and consideration of Chinese National Health Service Survey. CJHIM.

[20] Xu L, Wang Y, Collins CD (2007). Urban health insurance reform and coverage in China using data from National Health Services Surveys in 1998 and 2003. BMC Health Serv Res.

[21] Qun M, Ling X, Yaoguang Z (2012). Trends in Access to Health Services and Financial Protection in China between 2003 and 2011: A Cross-Sectional Study. Lancet.

[22] Wang M, Luo X, Xu S (2019). Trends in Smoking Prevalence and Implication for Chronic Diseases in China: Serial National Cross-Sectional Surveys from 2003 to 2013. Lancet Respir Med.

[23] Kim H-J, Jang J, Park E-C (2019). Unmet healthcare needs status and trend of Korea in 2017. Korean J Health Policy Manag.

[24] Zhou S, Huang T, Li A, Wang Z (2021). Does universal health insurance coverage reduce unmet healthcare needs in China? Evidence from the National Health Service Survey. Int J Equity Health.

[25] National Bureau of Statistics. Statistical system and classification standard (17). National Bureau of Statistics; 2022. http://www.stats.gov.cn/tjzs/cjwtjd/201308/t20130829_74318.html. Accessed 11 Feb 2023.

[26] Yuan B, Balabanova D, Gao J, Tang S, Guo Y (2019). Strengthening public health services to achieve universal health coverage in China. BMJ..

[27] National Center for Health Statistics. Health, United States, 2020–2021: People with hospital stays in the past year: United States, 1997–2019. National Center for Health Statistics; 2022. https://www.cdc.gov/nchs/hus/data-finder.htm. Accessed 20 Feb 2023.

[28] OECD. Hospital discharge rates (indicator). OECD; 2023. https://data.oecd.org/healthcare/hospital-discharge-rates.htm. Accessed 13 Feb 2023.

[29] Xie X, Hu Y (2022). The Reimbursement Rate of New Rural Cooperative Medical Scheme and Self-Rated Health Among Rural Middle-Aged and Elderly. Front Public Health..

[30] Hou Z, Van de Poel E, Van Doorslaer E (2014). Effects of NCMS on access to care and financial protection in China. Health Econ.

[31] Lu J, Lu Y, Wang X (2017). Prevalence, awareness, treatment, and control of hypertension in China: data from 1·7 million adults in a population-based screening study (China PEACE Million Persons Project). Lancet.

[32] Wang L, Gao P, Zhang M (2017). Prevalence and Ethnic Pattern of Diabetes and Prediabetes in China in 2013. JAMA.

[33] Li Y, Teng D, Shi X (2020). Prevalence of diabetes recorded in mainland China using 2018 diagnostic criteria from the American Diabetes Association: national cross sectional study. BMJ..

